# Concordance between Plasma and Filter Paper Sampling Techniques for the Lymphatic Filariasis Bm14 Antibody ELISA

**DOI:** 10.3390/tropicalmed2020006

**Published:** 2017-04-07

**Authors:** Jesse Masson, Jan Douglass, Maureen Roineau, Khin Saw Aye, Kyi May Htwe, Jeffrey Warner, Patricia M. Graves

**Affiliations:** 1 College of Public Health, Medical and Veterinary Sciences, James Cook University, Cairns, QLD 4870, Australia; jan.douglass@my.jcu.edu.au (J.D.); Maureenroineau@wanadoo.fr (M.R.); jeffrey.warner@jcu.edu.au (J.W.); patricia.graves@jcu.edu.au (P.M.G.); 2 Department of Medical Research, Myanmar Ministry of Health and Sports, Nay Pyi Taw, Myanmar; ksadmr@gmail.com (K.S.A.); Kyimaywin31@gmail.com (K.M.H.)

**Keywords:** lymphatic filariasis, Bm14, filter paper, DBS, CELISA, CDC

## Abstract

Diagnostic testing for the antibody Bm14 is used to assess the prevalence of bancroftian and brugian filariasis in endemic populations. Using dried blood spots (DBS) collected on filter paper is ideal in resource-poor settings, but concerns have been raised about the performance of DBS samples compared to plasma or serum. In addition, two versions of the test have been used: the Bm14 CELISA (Cellabs Pty Ltd., Manly, Australia) or an in-house CDC version. Due to recent improvements in the CELISA, it is timely to validate the latest versions of the Bm14 ELISA for both plasma and DBS, especially in settings of residual infection with low antibody levels. We tested plasma and DBS samples taken simultaneously from 92 people in Myanmar, of whom 37 (40.2%) were positive in a rapid antigen test. Comparison of results from plasma and DBS samples demonstrated no significant difference in positive proportions using both the CELISA (46.7% and 44.6%) and CDC ELISA (50.0% and 47.8%). Quantitative antibody unit results from each sample type were also highly correlated, with coefficients >0.87. The results of this study demonstrate that DBS samples are a valid collection strategy and give equivalent results to plasma for Bm14 antibody ELISA testing by either test type.

## 1. Introduction

Screening assays used in The Global Program to Eliminate Lymphatic Filariasis (GPELF) are frequently performed in low resource settings where samples may be exposed to temperature changes during collection, storage and transport. Diagnostic tests that can utilise dried blood spots (DBS) collected on filter paper which do not require immediate cold storage have many advantages over plasma samples in rural communities where lymphatic filariasis (LF) occurs.

The parasite antibody Bm14 diagnostic ELISA is among the techniques used to identify bancroftian- and brugian-associated lymphatic filariasis (LF) [1,2,3,4,5] and is used alongside antigen detection tests to monitor and evaluate endemicity in populations [2,6,7]. However, concerns over the specificity and predictive values of the Bm14 ELISA have raised questions about its accuracy in detecting residual endemicity or resurgence [8,9]. Of the three species detected by the Bm14 antibody test, *Wuchereria bancrofti* and *Brugia malayi* contribute 90% and 9% of LF disease burden worldwide [10,11,12].

The Bm14 ELISA, commercially produced as the CELISA (Cellabs Pty Ltd., Manly, Australia), incorporates positive and negative control samples to check plate to plate consistency [13]. The kit recommends using an optical density cut-off value for defining a positive result (0.4) [13], although some studies use a lower cut-off of 0.25 [10]. The CDC Bm14 in-house version of the assay recommends use of a standard curve to generate antibody units, with sample antibody values greater or equal to the cut-off being considered positive [14]. For comparability in the current study, we used standard curves generated from the same positive serum for both CELISA and CDC versions. Both versions of the Bm14 ELISA recommend the application of either plasma or eluted DBS for antibody detection.

While there is not a true gold standard for LF positivity, previous studies have found that the Bm14 test with plasma or serum has high sensitivity compared to microfilaria microscopy, recorded at 98% for samples from people with *W. bancrofti* and 91% for *Brugia malayi* filariasis [10]. Gass et al. [9] found similar high specificity of 95% for plasma in anticoagulant ethylenediaminetetraacetic acid (EDTA). However, CELISA DBS sensitivity can range from 50.0% to 92.3% when compared with immunochromatographic testing and PCR testing, with consistently high negative predictive values (NPV) of >96.6% [10].

While the dried blood spot method would be an inexpensive and convenient alternative to plasma samples, Joseph and colleagues [8] have reported that specificity of DBS when using the CELISA may be as low as 77%, with a positive predictive value (PPV) of 60% when compared to plasma application. Knowledge gaps in the literature and the release of an improved CELISA kit by Cellabs require additional evaluation and comparison of these methods.

This study investigates whether results obtained using DBS are valid and comparable to results obtained using plasma with the CELISA and the CDC ELISA. These results will provide confidence in and promote appropriate application of the Bm14 ELISA for whichever collection method is available.

## 2. Materials and Methods

### 2.1. Study Population

Amarapura Township within the Mandalay Region of Central Myanmar was selected as a study site, as it was known from sentinel site records to have a high prevalence of LF infection. All young people aged 10–21 years were invited to participate in a cross-sectional study. Ethical approval for the study was given by the Myanmar Ministry of Health and Sports and James Cook University Research Human Ethics Committee, approval number H5261.

Individuals were screened for LF infection using the BinaxNOW^®^ filariasis immunochromatographic test (ICT) card (Alere International Limited, Galway, Ireland). Young adults aged 18–21 years gave written consent to participate, while parents or guardians gave written consent for participants aged 10–17 years. An equal number of positive and negative participants were invited for a follow-up visit; however, some chose not to attend, resulting in a final collection of 37 ICT positives and 55 ICT negatives out of 92 total participants. The study sampling occurred shortly before the filariasis mass drug administration was offered in this township. Follow-up visits were done after the mass drug administration to ensure that positive participants took deworming drugs.

### 2.2. Preparation of Plasma and Blood Filter Paper Samples

Blood samples were collected by technical staff from the Myanmar Ministry of Health and Sports. A 10 mL sample of venous blood was collected from all participants and stored in cooled EDTA anticoagulant vacutainers (BD biosciences, Becton, Dickinson and Company, North Ryde, NSW, Australia). An amount of 10 μL of collected blood was transferred using a micropipette to each of the six protrusions of a filter paper disc (TropBio filter papers) and left to dry completely before storage in individual plastic bags. Remaining whole blood was kept on crushed ice and delivered to the Public Health Laboratory in Mandalay within four hours of collection. Plasma was separated from whole blood by centrifugation at 3000× *g* for 15 min and aliquoted into 2 mL tubes. Plasma samples were stored at −20 °C at the laboratory in Mandalay until transported to Yangon on dry ice and stored at −80 °C by the Department of Medical Research (DMR). One vial of each plasma sample was thawed, aliquoted and refrozen for transport to James Cook University in Cairns, Australia, where it was stored at −80 °C. Filter papers were sealed in plastic containers and kept in either 4 °C refrigeration or hand luggage during direct transport to Cairns, where they were stored at −80 °C.

### 2.3. Preparation of Eluates from DBS

DBS eluates were prepared for the application of the commercially available Bm14 kit (CELISA) [13] and the LF Bm14 CDC TMB-ELISA [14], using respective protocols. Sample diluent was prepared according to the instructions, with individual 495 μL and 245 μL sample diluent aliquots transferred into separate serum tubes for CELISA and CDC ELISA testing respectively, using a micropipette. Single blood-soaked filter paper protrusions were transferred to two separate serum tubes, one containing 495 μL of sample diluent to create 1:100 dilutions intended for CELISA application, and another containing 245 μL sample diluent to create a 1:50 dilution intended for CDC ELISA application. Each protrusion soaks exactly 10 μL of blood, with half of this volume estimated to be plasma and the remaining 5 μL containing all other blood products. Therefore, it is assumed that the 5 μL of plasma from each DBS added to 495 μL and 245 μL aliquots creates dilutions of 1:100 and 1:50 respectively. All samples were vortexed to ensure complete saturation of each disc and left to elute overnight at 4 °C before being warmed to room temperature (RT) of between 20 °C and 25 °C and vortexed again prior to testing.

### 2.4. Bm14 Filariasis Cellabs Enzyme Linked Immunosorbent Assay

Primary sample incubation was at 37 °C for 1 h before emptying and flooding wells with washing buffer three times, followed by a final emptying and upside down tapping of each plate to ensure wells were free of large droplets. Secondary IgG_4_ conjugates were added and incubated for 45 min at 37 °C, before washing again and applying a final 15-min incubation with tetramethylbenzidine (TMB) substrate without light exposure. Plates were prepared for optical density reading through the addition of 50 μL of stopping solution per well.

### 2.5. Bm14 Filariasis CDC’s Enzyme Linked Immunosorbent Assay

Antigen sensitising buffer (ASB) was created at 0.1 M using NaHCO_3_ and dH_2_O with a pH of 9.6 using NaOH. Working antigen solution (Atlanta, CDC) was prepared at 2 µg/mL using ASB. The binding of antigens to each well of Immulon 4HB plates (Thermofisher, Loughborough, UK) was achieved by adding 50 μL of 1:50 antigen solution to each well of a 96-well microplate before incubating at 4 °C overnight for at least 12 h. Working antigen solution was physically removed before the addition of 100 μL of PBS + 0.3% Tween (Life Technologies, Mulgrave, VIC, Australia), pH 7.2, to each well and incubated at 4 °C for 1 h.

Blanks were created by adding 50 μL of PBS with 0.05% Tween to intended blank wells. Dilutions of plasma or DBS eluates were added at 50 µL to each experimental well before incubating at room temperature on a rocker platform for 2 h. Washing steps between incubations were performed in an identical fashion to the CELISA, referred to earlier. Following the addition of horseradish peroxidase conjugated anti-human IgG_4_ (mouse) (Life Technologies), made to a 1:500 dilution with PBS with 0.05% Tween, at 50 µL to each plate well, the plate was incubated at room temperature for 45 min. TMB substrate was added at 50 µL to each well and incubated at room temperature in the dark for 5 min before adding 50 µL of 1 M HCL.

### 2.6. Statistical Analysis

Optical density readings of each sample were measured at a dual wavelength of 450 nm/650 nm with a VersaMax™ ELISA microplate reader (Molecular Devices, Sunnyvale, CA, USA) using SoftMax Pro Software Version 6.4.1 (Molecular Devices, Sunnyvale, CA, USA) with background absorbance of sample diluent subtracted.A moderately-positive sample collected from an endemic area of Papua New Guinea was used as a positive control, while negative controls were taken from Australian lab workers.

A standard curve was constructed using a single highly-positive sample from Papua New Guinea which was defined by an arbitrary high value of 1000 antibody units with subsequent 2-fold dilutions. A single cut-off point of >125 units was used to determine positive readings based on previous literature [5,9]. It should be noted that an optical density cut-off value of 0.4 is normally recommended by the CELISA manual. Relative sensitivity, specificity, positive predictive values (PPV), and negative predictive values (NPV), as well as paired *t*-tests, odds ratios, McNemar chi-square tests and correlations were performed using the IBM statistical software SPSS Version 23.0. Confidence intervals (CI) were reported at 95%.

## 3. Results

During October 2014, 377 young people residing in Amarapura Township were screened by ICT. Positive cases were age and gender matched to negative cases and 112 young people were invited to participate in a longitudinal study including provision of a blood sample. Not all participants returned for participation, leaving a final sample of 37 (40.2%) positive and 55 (59.8%) negative samples that were included in this study (Table 1).

### 3.1. Categorical Analysis and Comparisons of Plasma and DBS Using Cellabs and CDC ELISA Assays

Holding all CELISA plasma samples tested at a 1:100 dilution as a standard, we compared how DBS application affects the proportion of positive results when using the CELISA at an identical dilution. We found no significant difference in positive proportions between plasma (47%) and DBS (45%) samples (Table 1).

The CELISA standard was also compared to the CDC version of the Bm14 ELISA to assess any similarity in positive proportions when using plasma or DBS at the CDC recommended 1:50 dilution. The proportions of samples classed as positive were not significantly different between the CELISA standard (47%) and CDC ELISA with plasma (50%) or DBS (48%), respectively (Table 1). These three comparisons suggest that high similarity in positive results is found between the CELISA and the CDC ELISA using either plasma or DBS.

To establish how the CDC ELISA is affected by the application method, plasma and DBS samples were compared using the 1:50 dilution. Positive proportions of 50% when using plasma, and 48% when using DBS were not significantly different (Table 2). This confirms that the CDC ELISA will yield similar results regardless of the sample application method.

### 3.2. Comparing the Plasma and DBS for CELISA and CDC ELISA Samples

To determine how application of plasma and filter paper affects CELISA agreement, relative sensitivity, specificity, PPV and NPV were calculated. When DBS was compared to plasma at a 1:100 dilution using the CELISA, results were high overall at 88.4% sensitivity, 93.9% specificity, 92.7% PPV, and 90.2% NPV (Table 3). These results suggest that the CELISA yields reliable agreement between plasma and DBS samples.

Performance of the CDC ELISA using plasma and DBS at a 1:50 dilution was also compared to the CELISA 1:100 plasma standard. Relative sensitivity was high at 95.4% for both CDC plasma and DBS samples, with specificity found at 89.8% and 93.9% respectively (Table 3). Predictive values were also high for both CDC plasma and DBS samples compared to CELISA plasma, yielding 89.1% and 93.2% respectively for PPV, and 95.7% and 95.8% respectively for NPV (Table 3). Therefore, there is good agreement between the CDC ELISA using plasma and DBS when compared to results yielded by the CELISA using plasma.

Again, we compared plasma and DBS using the CDC ELISA at the recommended dilution of 1:50, to determine how test performance and relative predictive values are affected. Sensitivity and specificity were 97.7% and 93.8% respectively, while PPV and NPV were 93.5% and 97.8% respectively (Table 4). These results suggest that the CDC ELISA performs equally well when using either plasma or DBS, as confirmed by a high odds ratio value (Table 4).

### 3.3. Comparing Difference in Bm14 Antibody Units between Cellabs and CDC Assays

**Figure 1:**
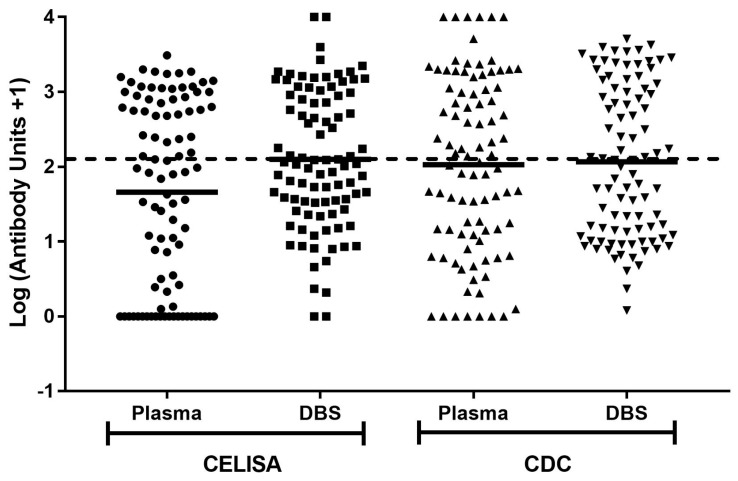
Filariasis Bm14 ELISA log (antibody unit concentration +1) value comparisons between plasma and DBS application of CELISA and CDC ELISA assays. Horizontal black lines refer to mean concentration of each assay, with the segmented line referring to the cut-off value at log (125 units +1). All concentrations above or equal to the defined cut-off are considered positive, while all readings below are considered negative.

All unit concentrations, with the addition of one to include results of zero, were converted to log values to approximate a normal distribution (Figure 1). Results were compared using geometric means. When analysing CELISA samples, the mean log value for plasma at 1.66 was significantly lower than the mean log value of 2.10 for DBS (*p* < 0.0001) (Table 5). However, the mean log values for CDC ELISA plasma and DBS samples at 2.03 and 2.07 respectively were not significantly different (*p* = 0.34) (Table 5). The data suggests a rise in estimated concentration when using DBS in comparison to plasma when using the CELISA, but not when using the CDC ELISA.

### 3.4. Correlation Coefficient Analysis of Bm14 Antibody Concentrations from Plasma and Eluates from Filter Paper

**Figure 2:**
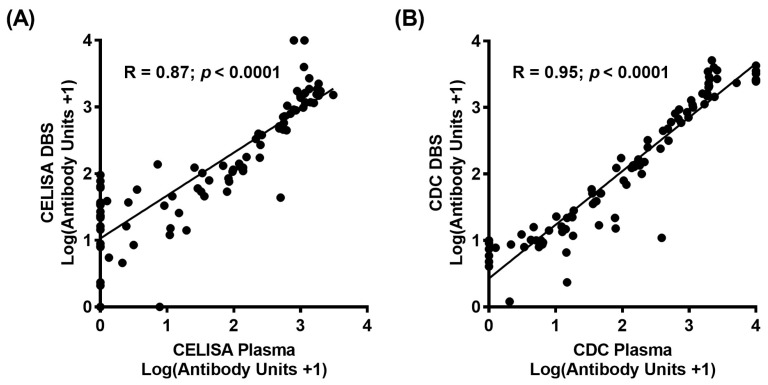
Filariasis Bm14 ELISA antibody unit concentration comparisons of plasma and whole blood application. (**A**) CELISA at 1:100 dilution comparisons of plasma against DBS; (**B**) CDC ELISA at 1:50 dilution comparisons of plasma against DBS.

The log values of Bm14 unit concentrations, with the addition of one to include results of zero, between plasma and DBS application were compared to determine correlation coefficients for both CELISA and CDC ELISA assays. Strong correlations were found at 0.87 and 0.95 for CELISA (Figure 2A) and the CDC ELISA (Figure 2B) respectively when comparing plasma and DBS samples (*p* < 0.0001). This shows that quantitative values between plasma and DBS are strongly positively associated when using the CELISA and the CDC ELISA.

## 4. Discussion

The major finding of this study is the strong agreement between plasma and DBS samples, taken from the same individuals and stored identically, when applied to either the Cellabs produced CELISA or the CDC Bm14 ELISA. In the Cellabs CELISA protocol [13], the recommended dilution is set at 1:100. According to the instructions for the in-house CDC filariasis serology assay [14], the recommended dilution is set at 1:50 dilution using serum obtained from centrifuged blood. Despite these differences in dilution optimised for the two tests, the proportions of samples classified as positive were not significantly different, with performance (relative sensitivity, specificity and predictive values) being very similar between all the tests.

High specificity and PPV values were found for comparisons of DBS against plasma using both CELISA and CDC ELISA tests. According to Joseph and Melrose [8], results obtained using the CELISA assay for DBS yielded a specificity of 77%, with 16 samples testing positive by DBS but negative by plasma, while PPV was found at 60%, suggesting that DBS sampling may result in false positives at an approximate rate of 40%. However, this work was done with an earlier version of the CELISA. Weil et al. [10] stated that blood samples with a known amount of antibody applied to DBS are not significantly different to those from serum samples under controlled conditions when using the CELISA. Our own results of 93.9% and 92.7% for specificity and PPV when comparing DBS and plasma suggest that the CELISA assay has improved in these regards.

Analysis of quantitative data showed that correlation was high between plasma and DBS for both the CELISA and CDC ELISA at >0.87. Although the mean antibody units were lower for DBS than plasma in the CDC ELISA, the difference was not significant. The significantly higher sample concentrations for DBS than plasma when using the CELISA suggests that this test gives higher background for DBS. However, the categorical results suggest no significant difference in agreement (classification of positives).If the amount of antibody present is important in future studies using DBS, it may be necessary to keep this difference in mind.

The use of DBS could facilitate more effective sample collection in endemic countries, where large-scale sampling must be undertaken with limited resources and also eliminates the risk of thawing or leaking during shipping.

Our analysis showed that both plasma and filter paper demonstrate similar results using both the CELISA and CDC ELISA. High agreement was also found when comparing the CELISA using plasma with CDC ELISA applications of either plasma or DBS. These results support the use of the Bm14 ELISA in assessing LF prevalence in the GPELF and can be used with either plasma or DBS on filter paper.

## Figures and Tables

**Table 1:** Positive proportions and chi-square results obtained when using the CELISA with plasma as a standard.

Standard	*N* Positive	% Positive (95% Confidence Interval (CI))	Comparative Test	*N* Positive	% Positive (95% CI)	McNemar Chi sq	*P* Value
CELISA Plasma 1:100	43	47 (37–57)	CELISA Dried blood spot (DBS) 1:100	41	45 (35–55)	0.5	0.7
-	-	-	CDC Plasma 1:50	46	50 (40–60)	1.3	0.5
-	-	-	CDC DBS 1:50	44	48 (38–58)	0.2	0.9

**Table 2:** Positives proportions and chi-square results obtained when comparing plasma and DBS with the CDC ELISA.

Standard	*N* Positive	% Positive (95% CI)	Comparative Test	*N* Positive	% Positive (95% CI)	McNemar Chi sq	*P* Value
CDC Plasma 1:50	46	50 (40–60)	CDC DBS 1:50	44	48 (38–58)	1.0	0.6

**Table 3:** Sensitivity, specificity, positive predictive value (PPV), negative predictive value (NPV) and odds ratio statistics comparisons of plasma and DBS application using the CELISA and CDC ELISA.

Standard	Comparative Test	Sensitivity (95% CI)	Specificity (95% CI)	PPV (95% CI)	NPV (95% CI)	Odds Ratio (95% CI)	*P* Value
CELISA Plasma 1:100	CELISA DBS 1:100	88.4% (74.9–96.1)	93.9% (83.1–98.7)	92.7% (80.8–95.5)	90.2% (80.1–95.5)	116.5 (26.15–519.4)	<0.0001
-	CDC Plasma 1:50	95.4% (84.2–99.4)	89.8% (77.8–96.6)	89.1% (78.1–95.0	95.7% (85.0–98.8)	180.4 (33.2–981.7)	<0.0001
-	CDC DBS 1:50	95.4% (84.2–99.4)	93.9% (83.1–98.7)	93.2% (82.0–97.6)	95.8% (85.6–98.9)	314.3 (50.0–1975.4)	<0.0001

**Table 4:** Sensitivity, specificity, PPV, NPV and odds ratio statistics comparisons of plasma and DBS application using the CDC ELISA.

Standard	Comparative Test	Sensitivity (95% CI)	Specificity (95% CI)	PPV (95% CI)	NPV (95% CI)	Odds Ratio (95% CI)	*P* Value
CDC Plasma 1:50	CDC DBS1:50	97.7% (88.0–99.9)	93.8% (82.8–98.7)	93.5% (82.7–97.7)	97.8% (86.6–99.7)	645.0 (64.6–6442.6)	<0.0001

**Table 5:** Geometric mean concentration values and *t*-test comparisons between plasma and DBS application of CELISA and CDC ELISA assays.

Group 1			Group 2			*t*-Test			
Test	Mean log (Antibody unit +1)	Geometric mean	Test	Mean log (Antibody unit +1)	Geometric mean	*N*	Mean Difference	SD	*p*
CELISA Plasma	1.66	45.00	CELISA DBS	2.10	124.42	92	0.44	0.63	<0.0001
CDC Plasma	2.03	105.62	CDC DBS	2.07	115.51	92	0.04	0.39	0.34
